# Supplementary material for: The Epigenetic Machinery and Energy Expenditure: A Network to Be Revealed
Source: Genes (Basel). 2025 Jan 19;16(1):104. doi: 10.3390/genes16010104 (PMC11764581; doi:10.3390/genes16010104)
Supplement: Supplementary file 1 [file genes-16-00104-s001.zip › genes-3365858-supplementary.pdf]

# Supplementary

**Supplementary Table S1.** The genetic defects of each patient of the cohort.

| Patient | Whole gene or exonic deletion | Mutation |
|---|---|---|
| **RSTS1 #1** | | *CREBBP* [NM_004380]: c.4633A>T; p.(Lys1545*) |
| **RSTS1 #2** | arr[hg19] 16p13.3(3845774_3927321)x1 | |
| **RSTS1 #3** | MLPA [kit P313]: deletion from the exon 14 to exon 31 in *CREBBP* gene | |
| **RSTS1 #4** | arr[hg19] 16p13.3(3504460_3984857)x1 | |
| **RSTS1 #5** | | *CREBBP* [NM_004380]: c.2616dupG; p.(Thr873Aspfs*97) |
| **RSTS1 #6** | | *CREBBP* [NM_004380]: c.4492C>T; p.(Arg1498*) |
| **RSTS1 #7** | | *CREBBP* [NM_004380]: c.4650_4654delAGAGA; p.(Glu1551Hisfs*2) |
| **RSTS1 #8** | MLPA [kit MRC-Holland]: deletion from the exon 29 to the exon 31 in *CREBBP* gene | |
| **RSTS1 #9** | MLPA [kit P313]: deletion from the exon 24 to the exon 31 in *CREBBP* gene | |
| **RSTS1 #10** | | *CREBBP* [NM_004380]: c.3524A>G; p.(Tyr1175Cys) |
| **RSTS1 #11** | | *CREBBP* [NM_004380]: c.223C>T; p.(Arg75*) |
| **SS1 #1** | | *NSD1* [NM_022455.4]: c.4792C>T; p.(Gln1597*) |
| **SS1 #2** | arr[hg19] 5q35.2q35.3(175719197_176883275)x1 | |
| **SS1 #3** | | *NSD1* [NM_022455.4]: c.3958C>T; p.(Arg1320*) |
| **SS1 #4** | | *NSD1* [NM_022455.4]: c.1527delT; p.(Ser510Valfs*2) |
| **SS1 #5** | | *NSD1* [NM_022455.4]: c.1810C>T; p.(Arg604*) |
| **SS1 #6** | | *NSD1* [NM_022455.4]: c.3958C>T; p.(Arg1320*) |

| | | |
|---|---|---|
| **SS1 #7** | | *NSD1* [NM_022455.4]: c.3958C>T; p.(Arg1320*) |
| **SS1 #8** | | *NSD1* [NM_022455.4]: c.1107delT; p.(Phe369Leufs*50) |
| **SS1 #9** | | *NSD1* [NM_022455.4]: c.2386_2389del; p.(Glu796Ilefs*10) |
| **SS1 #10** | | *NSD1* [NM_022455.4]: c.1633delA; p.(Thr545Argfs*10) |
| **WVS** | | *EZH2* [NM_004456.4]: c.2050C>T; p.(Arg684Cys) |
| **WDSTS #1** | | *KMT2A* [NM_001197104]: c.3294G>A; p.(Trp1098*) |
| **WDSTS #2** | | *KMT2A* [NM_001197104]: c.3461G>A; p.(Arg1154Gln) |
| **WDSTS #3** | | *KMT2A* [NM_001197104]: c.6873delG; p.(Met2291Ilefs16*) |
| **KMS1** | | *KMT2D* [NM_003482.3]: c.16295G>A; p.(Arg5432Gln) |
| **KLEFS1 #1** | | *EHMT1* [NM_024757.4]: c.3202T>A; p.(Cys1068Ser) |
| **KLEFS1 #2** | arr[hg19] 9q34.3 (140714395_140927911)x1 | |
| **KLEFS1 #3** | arr[hg19] 9q34.3 (140493556_141005513)x1 | |
| **KLEFS1 #4** | arr[hg19] 9q34.3 (140711171_140757122)x1 | |
| **KLEFS1 #5** | arr[hg19] 9q34.3 (140560792_141008915)x1 | |
| **KDVS** | arr[hg19] 17q21.13(43717703_44210822)x1 | |
| **SMS #1** | arr[hg19] 17p11 (17710076_17713445)x1 | |
| **SMS #2** | FISH [Kreatech]: deletion of the whole *RAI1* gene | |
| **CSS1 #1** | | *ARID1B* [NM_020732.3]: c.5049del; p.(Val1684Serfs*8) |
| **CSS1 #2** | | *ARID1B* [NM_020732.3]: c.6164G>A; p.(Trp2055*) |

| | | |
|---|---|---|
| **CSS1 #3** | | *ARID1B* [NM_020732.3]: c.3826G>T; p.(Glu1276*) |
| **FHLS** | | *SRCAP* [NM_006662]: c.7394delC; p.(Pro2465Glufs*10) |

**Supplementary Table S2.** Spearman correlation analyses between REE and biochemical variables for the overall cohort.

| | n | rho | p-value |
|---|---|---|---|
| **REE (kcal/die) and:** | | | |
| Prealbumin (microg/dL) | 35 | 0,05 | 0,79 |
| Leucine (microMol/L) | 27 | 0,17 | 0,41 |
| Insulin (ml/UL) | 32 | -0,11 | 0,56 |
| HDL (mg/dL) | 35 | 0,20 | 0,24 |
| ApoA I (mg/dL) | 33 | 0,13 | 0,46 |
| ApoA II (mg/dL) | 33 | 0,28 | 0,12 |
| ApoA (mg/dL) | 34 | 0,24 | 0,18 |
| ApoB (mg/dL) | 35 | -0,13 | 0,46 |
| Glycemia (mg/dL) | 35 | -0,04 | 0,80 |
| LDL (mg/dL) | 33 | 0,12 | 0,51 |
| **Prealbumin (microg/dL) and:** | | | |
| Leucine (microMol/L) | 29 | -0,21 | 0,28 |
| Insulin (ml/UL) | 35 | 0,38 | **0,02** |
| HDL (mg/dL) | 38 | -0,19 | 0,24 |
| ApoA I (mg/dL) | 36 | 0,08 | 0,64 |
| ApoA II (mg/dL) | 36 | 0,03 | 0,87 |
| ApoA (mg/dL) | 37 | -0,04 | 0,83 |
| ApoB (mg/dL) | 38 | 0,24 | 0,14 |
| Glycemia (mg/dL) | 38 | -0,06 | 0,74 |
| LDL (mg/dL) | 36 | 0,14 | 0,43 |
| **Leucine (microMol/L) and:** | | | |
| Insulin (ml/UL) | 27 | -0,10 | 0,64 |
| HDL (mg/dL) | 29 | 0,02 | 0,93 |
| ApoA I (mg/dL) | 28 | 0,13 | 0,52 |
| ApoA II (mg/dL) | 28 | 0,07 | 0,73 |
| ApoA (mg/dL) | 29 | 0,05 | 0,81 |
| ApoB (mg/dL) | 29 | 0,10 | 0,61 |
| Glycemia (mg/dL) | 29 | 0,06 | 0,77 |
| LDL (mg/dL) | 27 | 0,08 | 0,70 |
| **Insulin (ml/UL) and:** | | | |
| HDL (mg/dL) | 35 | -0,07 | 0,67 |
| ApoA I (mg/dL) | 33 | -0,01 | 0,94 |
| ApoA II (mg/dL) | 33 | -0,08 | 0,67 |
| ApoA (mg/dL) | 34 | 0,04 | 0,82 |
| ApoB (mg/dL) | 35 | 0,16 | 0,37 |
| Glycemia (mg/dL) | 35 | 0,32 | 0,06 |
| LDL (mg/dL) | 33 | 0,04 | 0,84 |
| **HDL (mg/dL) and:** | | | |
| ApoA I (mg/dL) | 36 | **0,73** | **<0,0001** |
| ApoA II (mg/dL) | 36 | 0,36 | 0,033 |
| ApoA (mg/dL) | 37 | **0,89** | **<0,0001** |
| ApoB (mg/dL) | 38 | 0,02 | 0,92 |
| Glycemia (mg/dL) | 38 | -0,13 | 0,43 |
| LDL (mg/dL) | 36 | 0,12 | 0,49 |
| **ApoA I (mg/dL) and:** | | | |
| ApoA II (mg/dL) | 35 | 0,22 | 0,20 |

| | | | |
|---|---|---|---|
| ApoA (mg/dL) | 35 | **0,82** | **<0,0001** |
| ApoB (mg/dL) | 36 | 0,15 | 0,38 |
| Glycemia (mg/dL) | 36 | -0,31 | 0,07 |
| LDL (mg/dL) | 34 | 0,19 | 0,28 |
| **ApoA II (mg/dL) and:** | | | |
| ApoA (mg/dL) | 36 | 0,40 | **0,016** |
| ApoB (mg/dL) | 36 | 0,30 | 0,07 |
| Glycemia (mg/dL) | 36 | 0,05 | 0,75 |
| LDL (mg/dL) | 34 | 0,37 | **0,033** |
| **ApoA (mg/dL) and:** | | | |
| ApoB (mg/dL) | 37 | 0,08 | 0,63 |
| Glycemia (mg/dL) | 37 | -0,08 | 0,63 |
| LDL (mg/dL) | 35 | 0,14 | 0,43 |
| **ApoB (mg/dL) and:** | | | |
| Glycemia (mg/dL) | 38 | -0,08 | 0,64 |
| LDL (mg/dL) | 36 | **0,81** | **<0,0001** |
